# The mediating role of the big five personality traits in the relationship between self-efficacy and depressive symptoms among Chinese university students

**DOI:** 10.3389/fpsyt.2025.1540216

**Published:** 2025-07-17

**Authors:** Yu Yang, Zhen Mou, Lingling Zhang, Shurong Ma, Zhenxiong Zhao

**Affiliations:** ^1^ School of Health Science and Engineering, University of Shanghai for Science and Technology, Shanghai, China; ^2^ College of Rehabilitation Sciences, Shanghai University of Medicine and Health Sciences, Shanghai, China; ^3^ Taizhou Central Hospital (Taizhou University Hospital), Taizhou, Zhejiang, China; ^4^ Tianjin Key Laboratory of Agricultural Animal Breeding and Healthy Husbandry, College of Animal Science and Veterinary Medicine, Tianjin Agricultural University, Tianjin, China; ^5^ Clinical Laboratory of Integrative Medicine, The First Affiliated Hospital of Dalian Medical University, Dalian, Liaoning, China

**Keywords:** depressive symptoms, self-efficacy, big five personality traits, university students, China

## Abstract

**Background:**

The mental health of university students, particularly depression, has become a significant public health concern in China. While previous studies have highlighted the link between self-efficacy and mental health, especially concerning depressive symptoms, the potential mediating role of the big five personality traits in this relationship remains underexplored. This study aims to examine the relationships among self-efficacy, the big five personality traits, and depressive symptoms through a mediation model.

**Methods:**

This cross-sectional study utilized a multi-stage stratified random sampling method to survey residents across 23 provinces in China, ultimately enrolling 1,193 university students aged 19-25. Measures included the PHQ-9 to assess depressive symptoms, the BFI-10 to evaluate personality traits, and the NGSES for self-efficacy. Hierarchical regression, random forest regression, mediation analyses, and restricted cubic spline (RCS) models were conducted using R software.

**Results:**

The prevalence of depressive symptoms among university students was 21.8%. Neuroticism (*p*<0.001) was a positive predictor of depressive symptoms, while agreeableness (*P*<0.001) and conscientiousness (*P*<0.001) were negative predictors. And agreeableness [Effect = -0.028, 95% CI (-0.045, -0.014)], conscientiousness [Effect = -0.043, 95% CI (-0.067, -0.023)], and neuroticism [Effect = -0.048, 95% CI (-0.070, -0.029)] significantly mediated the relationship between self-efficacy and depressive symptoms. Additionally, a potential nonlinear relationship (*p* for nonlinearity < 0.001) was identified between self-efficacy and depressive symptoms.

**Conclusions:**

Self-efficacy shows a direct positive association with depressive symptoms when controlling for personality traits, with neuroticism, agreeableness, and conscientiousness serving as key mediators. This highlights that the effect of self-efficacy on depression depends critically on personality, emphasizing the need to consider these traits in interventions for university students’ mental health.

## 1 Introduction

Depression is one of the most prevalent mental disorders worldwide, significantly impacting the quality of life and functioning of those affected. The World Health Organization estimates that 3.8% of the population experience depression, with this ratio expected to rise in the coming years (1). In China, the mental health of university students has also become a significant public health concern in recent years. The National Report on the Development of Mental Health (2021-2022) identifies university students as a high-risk group for depression. A survey of nearly 80,000 students found that the prevalence of depression among this population is 21.48% (2).

Depression poses a significant threat to the physical and mental health of university students, potentially leading to adverse outcomes such as reduced quality of life, poor academic performance, social difficulties, and functional impairment (3, 4). Furthermore, depression substantially increases the risk of suicide, which remains a leading cause of death among university students (5). As higher education expands and the number of university students grows, the mental health of this demographic not only has profound implications for individual development but also plays a crucial role in the social harmony and stability of society. Therefore, identifying the factors that influence depressive symptoms in this population and exploring the mechanisms underlying these influences are essential for effective prevention and intervention strategies.

Personality psychology provides an important framework for understanding individual differences in behavioral tendencies and emotional responses, with stable personality traits playing a significant role in mental health outcomes. The Five-Factor Model of Personality, which encompasses conscientiousness, agreeableness, neuroticism, openness, and extraversion, serves as a widely used framework for understanding personality (6). Drawing on the work of scholars such as Brown (7) and Kendler (8), Steunenberg et al. proposed that there is a well-established relationship between personality traits and depression, suggesting that a comprehensive model of depression’s etiology should incorporate all three types of influencing factors: personality, health-related factors, and social context (9). Personality traits play a significant role in influencing mental health among university students. Studies have shown that conscientiousness, agreeableness, extraversion, and openness—dimensions of the big five personality model—are generally negatively associated with depressive symptoms. In contrast, neuroticism, characterized by emotional instability and a tendency to experience negative emotions, is positively correlated with depression (10, 11). The university years represent a critical period of psychological and emotional development, during which individuals begin to form stable personality traits, establish self-identity, and lay the groundwork for future career development. Notably, neuroticism is among the most prevalent and severe personality traits associated with depression (12). It is defined by heightened emotional reactivity, elevated levels of anxiety, and a strong tendency toward negative affect (13), and is widely recognized as a major risk factor for depressive disorders (14, 15). These personality traits not only shape an individual’s emotional responses and behavioral tendencies but are also closely linked to their sense of self-efficacy.

Self-efficacy refers to an individual’s belief in their ability to successfully accomplish tasks and navigate challenges (16). As an essential internal psychological resource, self-efficacy has a strong relationship with personality traits as outlined in the big five framework (17). Evidence suggests that the lower the level of neuroticism and the higher the levels of extraversion, openness, agreeableness, and conscientiousness, the higher the general self-efficacy (18–20). Furthermore, self-efficacy plays a crucial role in managing stress, promoting family health, and preventing depressive symptoms among university students (21, 22). Although there is substantial evidence linking self-efficacy to depression, the role of personality traits, particularly the big five, remains insufficiently explored within this framework. Investigating how personality traits might mediate the relationship between self-efficacy and depressive symptoms is essential for gaining a more comprehensive understanding of these dynamics. Such insights could offer a more nuanced perspective on the interactions between personality, self-efficacy, and depression, particularly in the context of university students. Addressing these complex pathways is crucial not only for enhancing individual mental health but also for promoting broader societal well-being and development.

In this study, we hypothesize that higher self-efficacy will be associated with lower levels of depression (H1). Additionally, we hypothesize that the big five personality traits may mediate this relationship, with each trait (extraversion, conscientiousness, agreeableness, openness, and neuroticism) contributing uniquely to the pathway from self-efficacy to depression (H2). By examining these mediating mechanisms, the present study aims to deepen the understanding of how self-efficacy and personality traits interact to influence depression among university students. The findings may inform targeted interventions designed to enhance self-efficacy and address specific personality-related vulnerabilities, ultimately improving mental health outcomes in this population.

## 2 Methods

### 2.1 Study design

The data utilized in this study were derived from the Psychology and Behavior Investigation of Chinese Residents (PBICR) survey, conducted in China from July 10 to September 15, 2021, during the COVID-19 pandemic. This cross-sectional study employed a multistage sampling method. Based on data from the Seventh National Population Census of the People’s Republic of China in 2021, a sample of residents was selected from 120 cities using quota sampling, with quotas determined by gender, age, and urban-rural distribution. Participants were required to meet the following inclusion criteria: (1) holding Chinese nationality; (2) voluntarily participating in the study; (3) being able to independently complete the online questionnaire or with assistance from research personnel; and (4) being capable of understanding all items on the questionnaire.

From the initial 11,031 participants, 1,681 individuals aged 19 to 25 years who identified as students were selected. A total of 488 participants were excluded: 355 were either not currently enrolled in undergraduate education or were married, 70 had mental or physical disabilities (including visual, hearing, or speech impairments; reading or developmental disabilities; or chronic conditions such as hypertension), and 63 were taking medications during the survey period. The final analytic sample included 1,193 participants.

### 2.2 Measures

Basic demographic characteristics were measured, including gender, residence, household debt, living alone, body mass index (BMI). Additionally, the Patient Health Questionnaire-9 (PHQ-9) (23), the 10-item Big Five Inventory (BFI-10) (24), the New General Self-Efficacy Scale (NGSES) (25) were used to assess depressive symptoms, role of big five personality traits and self-efficacy, respectively. In this study, a PHQ-9 total score of ≥10 was considered as potentially indicating clinical depressive symptoms (26, 27). Moreover, all study variables were complete, with no missing data.

### 2.3 Statistical analysis

To present participants’ demographic characteristics, means ± standard deviations (SD) were provided for continuous variables, and independent t-tests and chi-square tests were utilized for comparing differences among groups. While for categorical variables, numbers (percentages) were presented, and Pearson chi-squared test was utilized to analyze inter-group differences. The bivariate correlation analysis was performed to examine the correlations among the study variables, including self-efficacy, big five personality traits, and depressive symptoms.

To evaluate the relationship between self-efficacy and depression, hierarchical regression models were constructed. Model 1 included self-efficacy and gender. Model 2 added residence, household debt, living alone, and BMI to the variables in Model 1. Model 3 further included the big five personality traits—openness, agreeableness, conscientiousness, extraversion, and neuroticism—on top of all covariates in Model 2. In the multicollinearity test, the variance inflation factor (VIF) (28) for each variable included in our analysis was determined, which was all below 2 (**Supplementary Table S1** **),** suggesting no evidence of significant multicollinearity. Subsequently, to investigate variable importance and interactions, a random forest regression model was employed to analyze the impact of self-efficacy and the big five personality traits on depressive symptoms. Hyperparameters were tuned using grid search to optimize model performance. Following, the mediating model was analyzed using the PROCESS macro. The model 4 (29), a simple mediation model, was used to examine whether there is a mediating effect of the big five personality traits on the relationship between self-efficacy and depression. The bias-corrected 95% confidence intervals (CI) were calculated through 5000 bootstrap resamples. If the 95% confidence interval of the indirect effect does not contain 0, it indicates a significant mediating effect. Otherwise, there is no mediating effect, with α = 0.05. Additionally, a fully adjusted multivariable restricted cubic spline (RCS) analysis (using 4 knots at the 5th, 35th, 65th, and 95th percentiles, respectively) was performed to assess the linearity and dose-response relationship between self-efficacy and depression. All statistical tests were two-tailed, with *p* < 0.05 considered statistically significant. And all statistical analyses were conducted using SPSS version 27.0 and R version 4.4.0.

## 3 Results

### 3.1 Sociodemographic characteristics and depressive symptoms

In this study, **Table 1** reports that a total of 1,193 participants were included, of whom 21.8% exhibited clinical depressive symptoms. Among the participants, 710 (59.51%) were female, 362 (30.34%) resided in urban areas, and 518 (43.42%) had household debt. In addition, 80 (6.71%) participants reported living alone. Notably, the differences of depressive symptoms were statistically significant (*p* < 0.05) in gender, living alone, openness, agreeableness, conscientiousness, extraversion, neuroticism, and self-efficacy, indicating that these factors have a significant impact on university students’ depressive symptoms.

**Table 1:** The demographic characteristics and the distribution of depressive symptoms.

Characteristics	Total	Depression status		p-value
		No	Yes	
n (%)	1193	933(78.2)	260(21.8)	
Gender				0.035
Male	483 (40.49)	363 (75.16)	120 (24.84)	
Female	710 (59.51)	570 (80.28)	140 (19.72)	
Residence				0.278
City	831 (69.66)	657 (79.06)	174 (20.94)	
Country	362 (30.34)	276 (76.24)	86 (23.76)	
Household debt				0.208
No	675 (56.58)	519 (76.89)	156 (23.11)	
Yes	518 (43.42)	414 (79.92)	104 (20.08)	
Living alone				<0.001
No	1113 (93.29)	414 (79.92)	104 (20.08)	
Yes	80 (6.71)	47 (58.75)	33 (41.25)	
BMI	20.93 ± 3.00	20.94 ± 2.98	20.92 ± 3.06	0.919
Openness	6.96 ± 1.48	7.02 ± 1.49	6.73 ± 1.42	0.004
Agreeableness	6.92 ± 1.36	7.04 ± 1.33	6.48 ± 1.38	<0.001
Conscientiousness	6.17 ± 1.38	6.26 ± 1.39	5.83 ± 1.28	<0.001
Extraversion	6.32 ± 1.64	6.36 ± 1.64	6.17 ± 1.65	0.085
Neuroticism	6.04 ± 1.42	6.14 ± 1.42	5.68 ± 1.35	<0.001
Self-efficacy	28.73 ± 5.46	28.94 ± 5.22	27.99 ± 6.20	0.024

Variables are presented as mean ± SD or n (%). BMI, body mass index.

### 3.2 Correlations of the studied variables

**Table 2** presents the correlations among self-efficacy, the big five personality traits, and depression symptoms in university students. The results indicate that self-efficacy is negatively correlated with depression symptoms (*r* = -0.059, *p* < 0.05) and neuroticism (r = -0.267, P < 0.01). Additionally, self-efficacy is positively correlated with openness (r = 0.198, P < 0.01), agreeableness (r = 0.165, P < 0.01), conscientiousness (r = 0.290, P < 0.01), and extraversion (r = 0.198, P < 0.01) of the big five personality traits. Furthermore, openness (r = -0.085, P < 0.01), agreeableness (r = -0.212, P < 0.01), conscientiousness (r = -0.151, P < 0.01), and extraversion (r = -0.104, P < 0.01) are all negatively correlated with depression symptoms. In contrast, neuroticism is positively correlated with depression symptoms (r = 0.204, P < 0.01).

**Table 2:** Correlations of the studied variables.

Variable	1	2	3	4	5	6
Depression						
Self-efficacy	-0.059*					
Openness	-0.085**	0.198**				
Agreeableness	-0.212**	0.165**	0.185**			
Conscientiousness	-0.151**	0.290**	0.125**	0.104**		
Extraversion	-0.104**	0.198**	0.201**	0.045	0.195**	
Neuroticism	0.204**	-0.267**	0.109**	0.224**	0.110**	0.201**

*p<0.05, **p<0.01.

### 3.3 Hierarchical regression analysis of predictive variables on depressive symptoms

**Table 3** presents the results of the hierarchical regression analysis. First, self-efficacy and gender were included in the model. Self-efficacy (*b* = -0.066, *p* = 0.038) had a significant effect on depressive symptoms, with an adjusted *R²* of 0.004. Next, residence, debt, living alone, and BMI were added to the model. Both self-efficacy (b = -0.063, *P =* 0.049) and living alone (b = 2.766, *P* = 0.001) significantly influenced depressive symptoms. At this stage, the adjusted R² increased from 0.004 to 0.014. Finally, when the five dimensions of the big five personality traits were introduced into the model, the effect of self-efficacy on depressive symptoms remained statistically significant (*P =* 0.035). Notably, the regression coefficient shifted from -0.063 to 0.070. Among the big five traits, neuroticism, conscientiousness, and agreeableness were significantly related to depressive symptoms. The adjusted *R²* increased from 0.014 to 0.097. Specifically, conscientiousness (b = -0.581, *P* < 0.001) and agreeableness (b = -0.670, *P* < 0.001) were negative predictors of depressive symptoms, while neuroticism (b = 0.705, *P* < 0.001) was a positive predictor.

**Table 3:** Hierarchical regression analysis of depressive symptoms.

Forecast variable	Model1			Model2			Model3		
	*β*	t	*p*	*β*	t	*P*	*β*	t	*P*
Self-efficacy	-0.066	-2.075	0.038	-0.063	-1.972	0.049	0.070	2.115	0.035
Gender	-0.526	-1.485	0.138	-0.416	-1.141	0.254	-0.573	-1.617	0.106
Residence				0.331	0.873	0.383	0.212	0.576	0.565
Household debt				-0.117	-0.335	0.738	-0.161	-0.479	0.632
Living alone				2.766	3.977	<0.001	2.641	3.942	<0.001
BMI				-0.011	-0.185	0.853	-0.028	-0.489	0.625
Openness							-0.081	-0.684	0.494
Agreeableness							-0.670	-5.19	<0.001
Conscientiousness							-0.581	-4.531	<0.001
Extraversion							-0.175	-1.628	0.104
Neuroticism							0.705	5.583	<0.001
F	3.178			3.922			12.580		
Adjusted R²	0.004			0.014			0.097		

### 3.4 Random forest regression analysis of predictive variables on depressive symptoms

**Figure 1:**
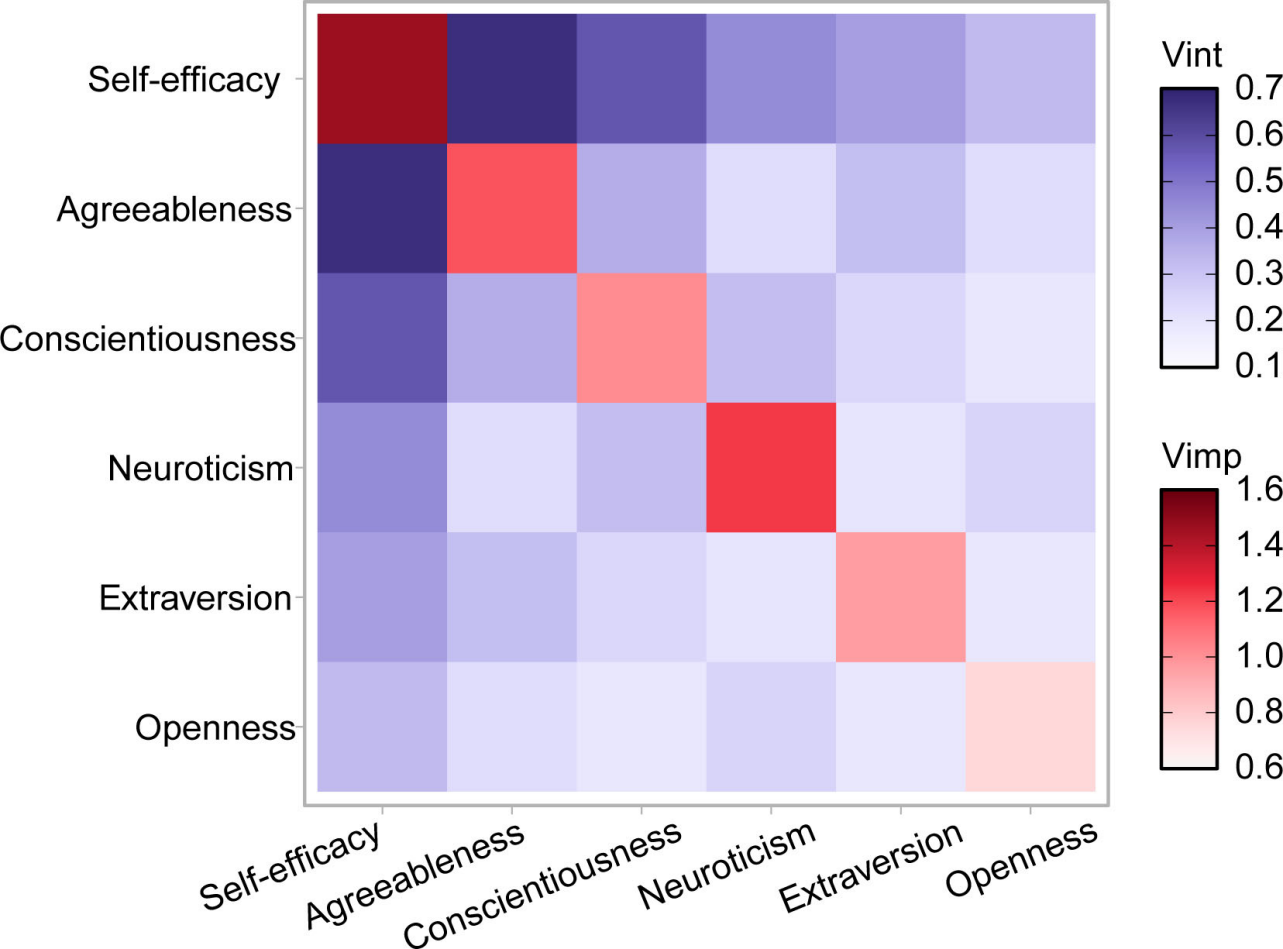
Variable importance and interaction heatmap for predictors of depression. Vint: The depth of color represents the interaction effect between variables, with darker colors indicating a stronger interaction effect (0.7 being the maximum value). Vimp: The depth of color represents the importance of each variable, with darker colors indicating a greater contribution to the model’s prediction of depression (1.6 being the maximum value).

The results of the random forest regression model indicate that (**Figure 1**), in terms of variable importance, self-efficacy is the most significant predictor in the model, with a variable importance (VImp) value of 1.47. This underscores its substantial impact on predictions. The following are neuroticism, agreeableness, and conscientiousness, with VImp values of 1.23, 1.175, and 1.019, respectively.

Regarding variable interactions, the interaction between self-efficacy and agreeableness is the most pronounced, with a variable interaction measure (VInt) value of 0.674. Next, the interaction between self-efficacy and conscientiousness reaches a value of 0.575. Other interactions, such as those between self-efficacy and neuroticism, extraversion, and openness, show values of 0.448, 0.404, and 0.33, respectively. These results highlight the importance of these variables and their interactions in predicting depression (**Supplementary Table S2**).

### 3.5 Mediation analysis of big five personality traits

**Figure 2:**
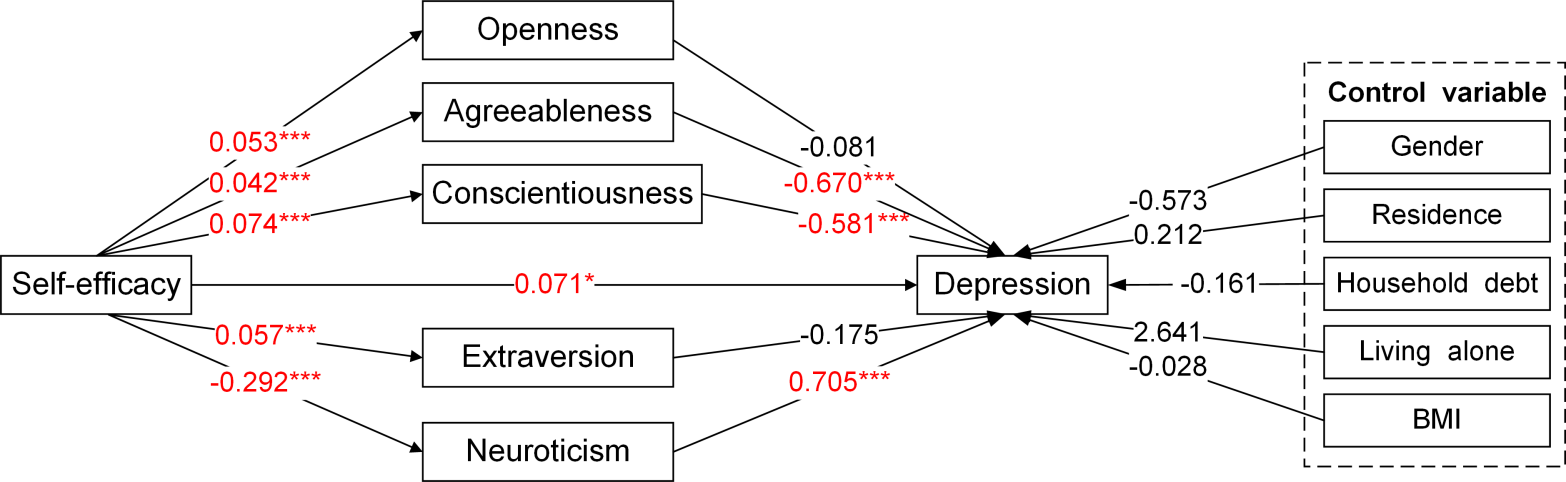
The intermediary verification model of big five personality. Controlling gender, residence, household delt, living alone, BMI. *p<0.05, ***p<0.001.

Adjusted mediation models controlling for variables (**Figure 2**) showed that self-efficacy was significantly associated with the five dimensions of the big five personality traits. Specifically, self-efficacy had a significant positive effect on openness (*b* = 0.053, *p* < 0.001), agreeableness (b = 0.042, P < 0.001), conscientiousness (b = 0.074, P < 0.001), and extraversion (b = 0.057, P < 0.001) and a significant negative effect on neuroticism (b = -0.292, P < 0.001).

**Figure 3:**
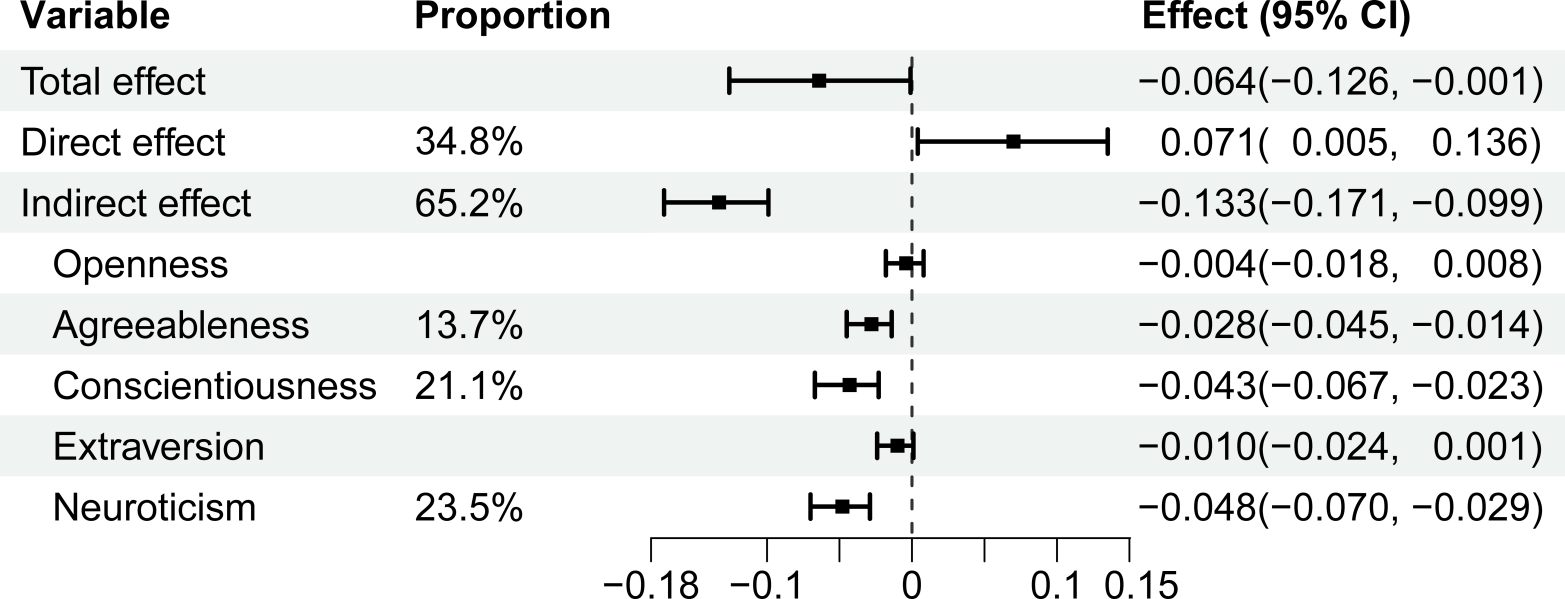
Mediation analysis of self-efficacy on depressive symptoms. $\frac{\left|M\right|}{\left|direct\;effect\right|+\left|indirect\;effect\right|}$ , M. agreeableness, conscientiousness, neuroticism .

Further mediation analysis (**Figure 3**) showed significant total and direct effects of self-efficacy on depressive symptoms, with effect sizes of [Effect = -0.064, 95% CI (-0.126, -0.001)] and [Effect = 0.071, 95% CI (0.005, 0.136)], respectively. Additionally, the mediation effect was statistically significant [Effect = -0.133, 95% CI (-0.171, -0.099)], accounting for 65.2% of the total effect. Notably, among the five mediation paths examined, only agreeableness [Effect = −0.028, 95% CI (−0.045, −0.014)], conscientiousness [Effect = −0.043, 95% CI (−0.067, −0.023)], and neuroticism [Effect = −0.048, 95% CI (−0.070, −0.029)] showed statistically significant mediation effects, accounting for 13.7%, 21.1%, and 23.5% of the total effect, respectively.

### 3.6 Trend analysis of self-efficacy on depressive symptoms

**Figure 4:**
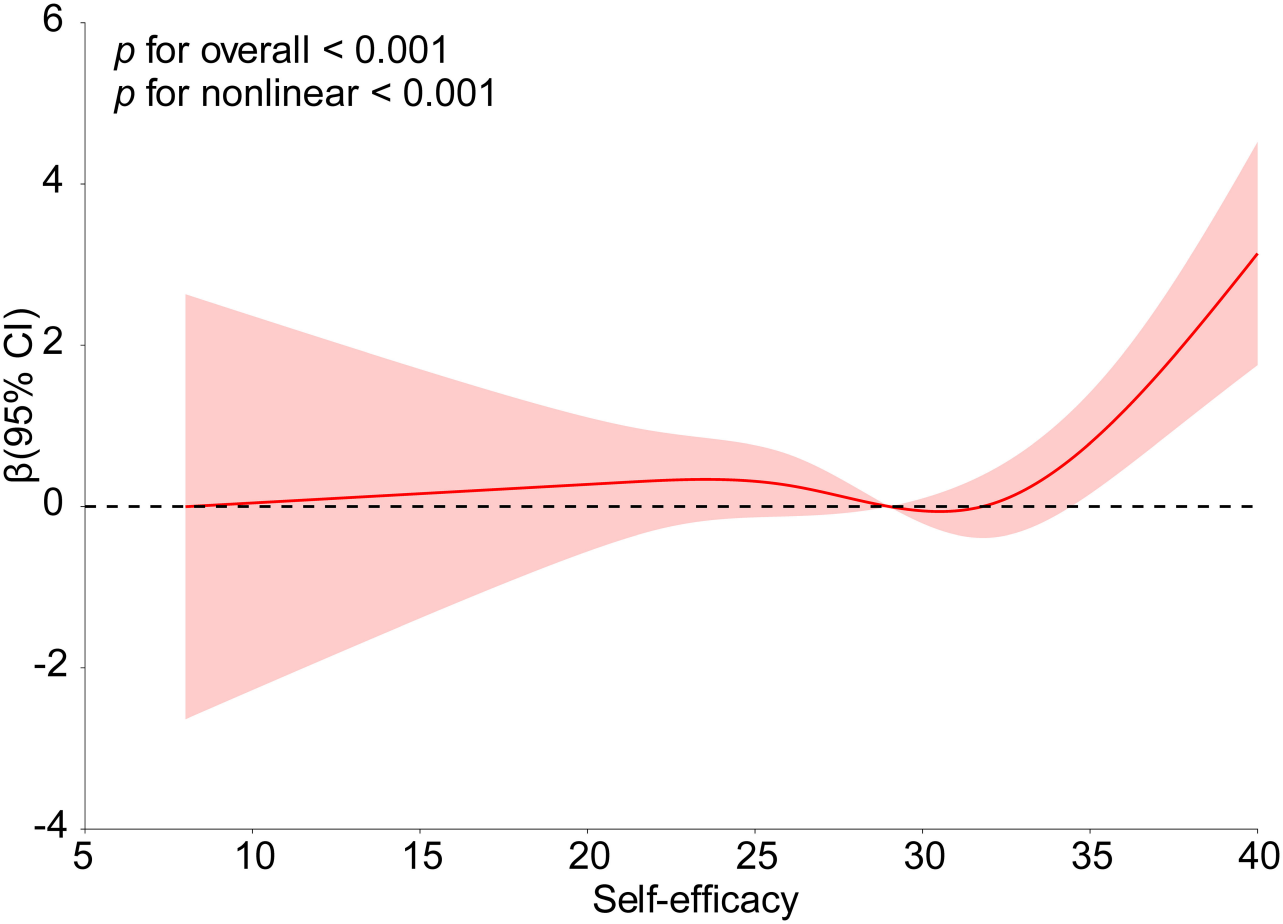
Association of between self-efficacy and depression. The model was adjusted for gender, residence, household delt, living alone and BMI.

Due to the differing trends between the direct effect of self-efficacy on depressive symptoms and its total and indirect effects observed in the mediation analysis, we further investigated the potential nonlinear relationship between self-efficacy and depressive symptoms using RCS curves. **Figure 4** illustrates a significant nonlinear relationship between self-efficacy and depressive symptoms (*p* for overall < 0.001, *P* for nonlinearity < 0.001).

## 4 Discussion

In this study, the prevalence of depressive symptoms among Chinese university students during the COVID-19 pandemic was 21.8%. This relatively high prevalence of depression is comparable to estimates from other related analyses, such as a recent study which found a 21.1% prevalence among Chinese university students (30). Similarly, the prevalence of depressive symptoms was reported to be 29% among French students (26), 25.3% at Capital Medical University (31), and 24.3% among medical students, with 30.6% experiencing generalized anxiety disorder (32). Regional differences and varying measurement methods may account for some of these discrepancies. Overall, the high incidence of depressive symptoms among university students during the COVID-19 pandemic warrants greater attention.

Although previous research has demonstrated a robust association between self-efficacy and depressive symptoms, the moderating role of personality traits in this relationship remains insufficiently explored, particularly among university students. In our linear regression analyses (Model 1 and Model 2), we observed a significant negative association between self-efficacy and depressive symptoms, thereby supporting Hypothesis H1 and aligning with prior findings (33). However, upon the inclusion of the big five personality traits in Model 3, the direction of the relationship reversed, with self-efficacy emerging as a positive predictor of depressive symptoms. This counterintuitive finding suggests the presence of a suppression effect (34), indicating that the link between self-efficacy and depression is more nuanced than initially assumed. The inclusion of personality variables in Model 3 served to control for the variance shared between self-efficacy and specific personality traits, thereby unveiling the heterogeneous components embedded within the self-efficacy construct. For instance, individuals high in neuroticism are typically characterized by elevated levels of anxiety and worry—key contributors to depressive symptoms (35). Once neuroticism is accounted for, the shared variance it holds with self-efficacy is partial led out, revealing potentially maladaptive facets of self-efficacy. At this point, self-efficacy may no longer solely represent confidence or effective coping, but instead reflect tendencies toward perfectionism, compulsive control, or unrealistic self-standards—features that may paradoxically increase vulnerability to depression (36). Moreover, some studies suggest that the relationship between self-efficacy and psychological outcomes may be non-linear. For example, Schönfeld and colleagues demonstrated that increases in self-efficacy do not invariably lead to reductions in stress or improved functioning, highlighting a complex, non-monotonic relationship (37). Further, existing literature has pointed out that self-efficacy is not uniformly beneficial; elevated self-efficacy can sometimes induce heightened neuroendocrine and psychological stress responses, and even undermine performance—an aspect that has been largely overlooked (38). Moores and Chang support this view, showing that while self-efficacy generally predicts improved performance in IT-related domains, excessive self-efficacy can result in overconfidence, which in turn negatively impacts subsequent performance (39). This finding suggests that the impact of self-efficacy may depend on an individual’s ability to accurately assess their own capabilities. When self-efficacy is inflated, it may hinder adaptive functioning and increase psychological stress when faced with challenges. Pintrich does not view self-efficacy as a static trait but rather as varying across different performance domains (40). This perspective is particularly relevant in the highly competitive (“involutional”) educational and sociocultural context of China, where some students may display outwardly high self-efficacy that is not grounded in genuine internal control beliefs, but rather driven by anxiety, compulsiveness, or perfectionistic strivings (41). Such “confidence” often resembles an aversion to failure rather than a healthy sense of self-efficacy. In striving to meet intense familial, academic, and societal expectations for “success,” these individuals may internalize excessively elevated or rigid beliefs about their abilities (42). Therefore, the reversal of the self-efficacy coefficient observed in this study is not merely a statistical artifact, but a theoretically meaningful insight: self-efficacy is neither stable nor uniformly protective. Its effects are likely shaped by the broader constellation of personality traits and specific psychological contexts. Under high-pressure, failure-intolerant cultural environments, self-efficacy may paradoxically function as a risk factor for depressive symptoms. These findings underscore the importance of examining the motivational substrates and personality dynamics that underlie self-efficacy, in order to more accurately understand its impact on mental health. Further research is thus warranted to delineate the mechanisms and boundary conditions of this complex relationship.

Building on this, we observed significant interaction effects between self-efficacy and three personality traits—agreeableness, conscientiousness, and neuroticism. These interactions exerted a negative influence on depressive symptoms, thereby offering a deeper explanation for why the regression coefficient for self-efficacy turned positive in Model 3. Notably, within the mediation analysis framework, the coefficient observed in Model 3 corresponds to the direct effect, which can be conceptualized as the total effect minus the indirect effects. These findings lend support to Hypotheses H2b, H2c, and H2e, and are consistent with previous research (43). Specifically, university students exhibiting high levels of conscientiousness are more likely to develop a strong sense of self-efficacy, maintain positive affect and outlook, and effectively manage various stressors and challenges in daily life, thereby lowering their susceptibility to depressive symptoms (44). Individuals with high agreeableness and conscientiousness are empathetic and cooperative, resulting in pleasant and harmonious relationships with others (45) An online study of 635 Finnish university students revealed that students with high agreeableness tend to have lower tendencies of rumination, self-reported stress, depressive symptoms, and anxiety (46). A cross-sectional study in South Korea also indicated that low agreeableness is associated with depression in young people (47). Furthermore, a meta-analysis of 13 longitudinal datasets (comprising 2,518 individuals and 11,170 momentary emotion assessments) found that individuals with high levels of neuroticism exhibited significantly greater fluctuations in negative affect in daily life (48). From a neurobiological perspective, agreeableness has been closely associated with increased activity in the temporoparietal junction (TPJ) and other brain regions involved in empathy and social cognition. Conscientiousness is linked to heightened activation in the prefrontal cortex, reflecting its role in self-regulation and goal-directed behavior. In contrast, neuroticism is typically associated with hyperactivity in the amygdala and other limbic system structures, indicating a heightened sensitivity to emotional stimuli and stress (49).

In our study, hypotheses H2a and H2d were not supported, indicating that openness and extraversion do not have a mediating effect. Previous research frequently found no significant correlation between openness and depression (50). Openness is divided into six narrower facets: fantasy, aesthetics, feelings, actions, ideas, and values (51). However, the relationships between these six dimensions and depression are not consistent, with only the aesthetic aspect positively correlated with depression, while the other five aspects are not significant (52). This inconsistency may explain the lack of a clear link between openness and depression, thereby suggesting that openness may not play a notable mediating role in the relationship between self-efficacy and depression. Extraversion, characterized by sociability, high energy levels, and an appreciation for social interactions (53), is typically associated with positive mental health outcomes and reduced symptoms of depression, anxiety, and psychological disorders (54, 55). However, in our study extraversion was negatively associated with depressive symptoms, although this association was not statistically significant. This discrepancy may be attributed to the unique context of the COVID-19 pandemic, during which social interactions were severely disrupted and exposure to negative media content increased dramatically. These factors may have attenuated the protective effects of extraversion. A study involving 486 adults during the pandemic revealed that the protective effect of extraversion against depression was associated with higher levels of social media use (SMU), which in turn was linked to poorer mental health outcomes, including increased depression and anxiety (56). This suggests that the relationship between extraversion and mental health is not straightforward but rather moderated by the extent of SMU. Future research should further explore the complex interplay between extraversion, openness, and depressive symptoms, especially in the context of evolving social and media environments.

These findings suggest that interventions addressing both personality traits and self-efficacy may be more effective in reducing depressive symptoms among university students. For individuals with high neuroticism scores, maladaptive cognitive patterns can be addressed and coping self-efficacy can be trained to change their coping strategies. Cognitive-behavioral therapy (CBT) is particularly effective in this regard (57). University counseling centers could offer group-based CBT sessions focusing on cognitive restructuring to challenge negative thoughts and behavioral activation to encourage engagement in positive activities. Additionally, online CBT modules could provide students with self-guided resources. A structured program could run for 8 to 12 weeks, with weekly sessions lasting 1 to 2 hours, covering key strategies such as reframing negative thoughts, setting achievable goals, and practicing relaxation techniques. Other approaches, such as acceptance and commitment therapy (ACT) and mindfulness meditation, have also been shown to reduce depressive symptoms (58, 59). Mindfulness practices could be integrated into student activity centers or online platforms, offering guided sessions on body scans, mindful breathing, and movement. Weekly sessions of 30 to 60 minutes, conducted one to two times per week, could help students cultivate present-moment awareness and manage stress more effectively. To support students’ mental well-being, university administrators, counseling centers, and student activity coordinators should implement these evidence-based programs. By fostering resilience and adaptive coping strategies, institutions can better equip students to handle stress and maintain psychological health.

## 5 Limitations

Firstly, this study employs a cross-sectional design. Although this design is well-suited to our research hypothesis, it does not permit us to draw causal inferences. Future research could utilize a longitudinal design to verify causality and accurately explore the threshold effects of self-efficacy and depression. Secondly, this study relies on self-reported data, which is a limitation as it increases the risk of social desirability bias. Incorporating qualitative research methods, such as in-depth interviews, can further explore the relationships between self-efficacy, personality, and depression, thereby enhancing the credibility of the results. Thirdly, the study sample is limited to Chinese university students, which may restrict the generalizability of the findings to other populations. Future research should expand the sample to include diverse demographic and cultural groups to examine whether the observed relationships hold across different contexts.

## 6 Conclusions

This study reveals a complex relationship between self-efficacy, personality traits, and depressive symptoms among Chinese university students during the COVID-19 pandemic. While self-efficacy is typically considered protective against depression, our mediation analysis indicates a counterintuitive direct positive association with depressive symptoms after controlling for personality traits. Notably, neuroticism, agreeableness, and conscientiousness significantly mediated this relationship, suggesting that the psychological impact of self-efficacy is contingent upon underlying personality dispositions. These findings underscore the importance of accounting for personality-based pathways when designing interventions targeting depressive symptoms in university populations.

## Data Availability

Publicly available datasets were analyzed in this study. This data can be found here: https://www.x-mol.com/groups/pbicr/research.
