# Supplementary material for: The mediating role of the big five personality traits in the relationship between self-efficacy and depressive symptoms among Chinese university students
Source: Front Psychiatry. 2025 Jul 17;16:1540216. doi: 10.3389/fpsyt.2025.1540216 (PMC12310589; doi:10.3389/fpsyt.2025.1540216)
Supplement: Supplementary file 1 [file Table1.docx]

| Table S1. Collinearity Statistics. | |
| --- | --- |
| Variables | GVIF |
| Self-efficacy | 1.217 |
| Gender | 1.107 |
| Residence | 1.043 |
| Household debt | 1.013 |
| Living alone | 1.026 |
| BMI | 1.079 |
| Openness | 1.117 |
| Agreeableness | 1.123 |
| Conscientiousness | 1.140 |
| Extraversion | 1.130 |
| Neuroticism | 1.168 |

| Table S2. Variable importance and interactions in predicting depression. | | | | | | |
| --- | --- | --- | --- | --- | --- | --- |
|  | Self-efficacy | Agreeableness | Conscientiousness | Neuroticism | Extraversion | Openness |
| Self-efficacy | 1.472 |  |  |  |  |  |
| Agreeableness | 0.674 | 1.175 |  |  |  |  |
| Conscientiousness | 0.575 | 0.364 | 1.019 |  |  |  |
| Neuroticism | 0.448 | 0.226 | 0.321 | 1.235 |  |  |
| Extraversion | 0.404 | 0.317 | 0.246 | 0.199 | 0.963 |  |
| Openness | 0.33 | 0.227 | 0.19 | 0.258 | 0.191 | 0.751 |
